# A causal association between immune cells and hypertrophic cardiomyopathy: A bidirectional Mendelian randomization study

**DOI:** 10.1016/j.gendis.2025.101539

**Published:** 2025-01-21

**Authors:** Fang He, Qiangzhong Pi, Jun Yang, Mengning Wan, Jiang Yu, Ding Yang, Yongzheng Guo, Xiaorong Li

**Affiliations:** a Division of Nephrology, The First Affiliated Hospital of Chongqing Medical University, Chongqing 400016, China; b Department of Respiratory Medicine, Southwest Hospital, Army Military Medical University, Chongqing 400038, China; c Division of Anesthesiology, The First Affiliated Hospital of Chongqing Medical University, Chongqing 400016, China; d Division of Cardiology, The First Affiliated Hospital of Chongqing Medical University, Chongqing 400016, China; e Cardiovascular Disease Laboratory of Chongqing Medical University, Chongqing 400016, China

Hypertrophic cardiomyopathy (HCM) is a prevalent inherited cardiac condition, affecting approximately 1 in 500 individuals.^1^ Recent research highlights immune cell involvement in HCM, with altered levels of various immune populations associated with the disease.^2^ However, whether these changes are causative or merely correlational is still uncertain. This study aims to investigate the causal effects of 731 immune cell types on HCM using comprehensive bidirectional Mendelian randomization (MR), a robust method for assessing causal inference in observational studies.^3^

Our overall workflow is displayed in Figure S1 and the detailed methods are shown in the Supplementary Methods. The brief information on the data included is shown in Table S1. Instrumental variables were selected from the immune cell GWAS summary statistics using a *P*-value threshold of 1 × 10^−5^ to ensure robust associations. We applied a cutoff for F statistics greater than 10 to mitigate bias from weak instrumental variables. Clumping based on linkage disequilibrium (R^2^ < 0.001 within a 1000-kb distance) was performed to ensure independence among single nucleotide polymorphisms (SNPs). We also excluded SNPs associated with confounders and outcomes using the PhenoScanner tool. To assess causal relationships, we employed inverse variance weighting and MR-Egger regression methods, with statistical significance set at *P* < 0.05. Cochran's Q statistic and I^2^ statistics were calculated to test for heterogeneity among SNPs. The MR-Egger intercept test was conducted to identify potential pleiotropy. Additionally, a leave-one-out analysis was performed to evaluate the influence of individual SNPs on the overall causal estimates.

Following rigorous screening, 16,958 SNPs were identified as associated with immune cells for the final HCM dataset (Table S2). The F-statistics ranged from 19.548 to 243.818 (mean = 33.665), indicating that the selected SNPs were unlikely to be biased by weak instrumental variables. In total, 17,831 SNPs from the immune cell dataset associated with HCM were also included, with F-statistics ranging from 19.611 to 327.573 (mean = 53.387). The details associated with the data are listed in Table S3.

The inverse variance weighting analysis revealed significant causal associations between 31 immune cell types and HCM (Fig. 1A). Among these, 19 immunophenotypes were identified as protective factors, including effector memory CD4^−^ CD8^−^ T cell %CD4^−^ CD8^−^ T cell (odds ratio/OR = 0.9601, *P* = 0.0079), effector memory CD8^+^ T cell %T cell (OR = 0.9188, *P* = 0.0471), HLA DR on monocyte (OR = 0.9156, *P* = 0.0497), CD11c^+^ HLA DR^++^ monocyte %monocyte (OR = 0.9126, *P* = 0.0489), CD45 on HLA DR^+^ T cell (OR = 0.9061, *P* = 0.0346), granulocyte %leukocyte (OR = 0.8959, *P* = 0.0374), HLA DR on CD14^+^ CD16^−^ monocyte (OR = 0.8915, *P* = 0.0124), CD3 on terminally differentiated CD8^+^ T cell (OR = 0.8900, *P* = 0.0471), CD86 on CD62L^+^ myeloid dendritic cell (OR = 0.8838, *P* = 0.0156), CD3 on CD39^+^ resting CD4 regulatory T cell (OR = 0.8829, *P* = 0.0163), HLA DR on CD14^+^ monocyte (OR = 0.8801, *P* = 0.0068), CD3 on CD28^−^ CD8^+^ T cell (OR = 0.8573, *P* = 0.0185), HLA DR^+^ CD4^+^ T cell %T cell (OR = 0.8428, *P* = 0.0179), IgD^+^ CD24^+^ B cell %B cell (OR = 0.8308, *P* = 0.0427), CD4^−^ CD8^−^ T cell %T cell (OR = 0.8136, *P* = 0.0342), SSC-A on lymphocyte (OR = 0.8076, *P* = 0.0275), unswitched memory B cell absolute count (OR = 0.8076, *P* = 0.0145), transitional B cell %lymphocyte (OR = 0.8051, *P* = 0.0025), and HLA DR^++^ monocyte %leukocyte (OR = 0.7899, *P* = 0.0064). Conversely, 12 immune cell types were recognized as risk factors for HCM, such as CD4^+^ CD8dim T cell %leukocyte (OR = 1.2546, *P* = 0.0362), CCR2 on CD62L^+^ myeloid dendritic cell (OR = 1.1761, *P* = 0.0181), HLA DR on CD14^−^ CD16^+^ monocyte (OR = 1.1731, *P* = 0.0012), CD8^+^ T cell %T cell (OR = 1.1327, *P* = 0.0479), CD62L^−^ myeloid dendritic cell %dendritic cell (OR = 1.1282, *P* = 0.0056), IgD on IgD^+^ CD38^−^ unswitched memory B cell (OR = 1.1233, *P* = 0.0140), CD62L^−^ CD86^+^ myeloid dendritic cell %dendritic cell (OR = 1.1096, *P* = 0.0306), IgD on unswitched memory B cell (OR = 1.0952, *P* = 0.0314), CD25 on B cell (OR = 1.0874, *P* = 0.0376), CD45 on B cell (OR = 1.0785, *P* = 0.0424), CD16^−^ CD56 on HLA DR^+^ natural killer (OR = 1.0722, *P* = 0.0265), and terminally differentiated CD4^−^ CD8^−^ T cell %CD4^−^ CD8^−^ T cell (OR = 1.0450, *P* = 0.0157). The results of heterogeneity and pleiotropy analyses (Table S4) supported the reliability of these findings, with no significant evidence suggesting confounding factors. The scatter plots and funnel plots in Figure S2 and Figure S3 demonstrate the stability of the above results. Additionally, the robustness of the causal associations was demonstrated in the results from sensitivity analysis in Figure S4.

**Figure 1:**
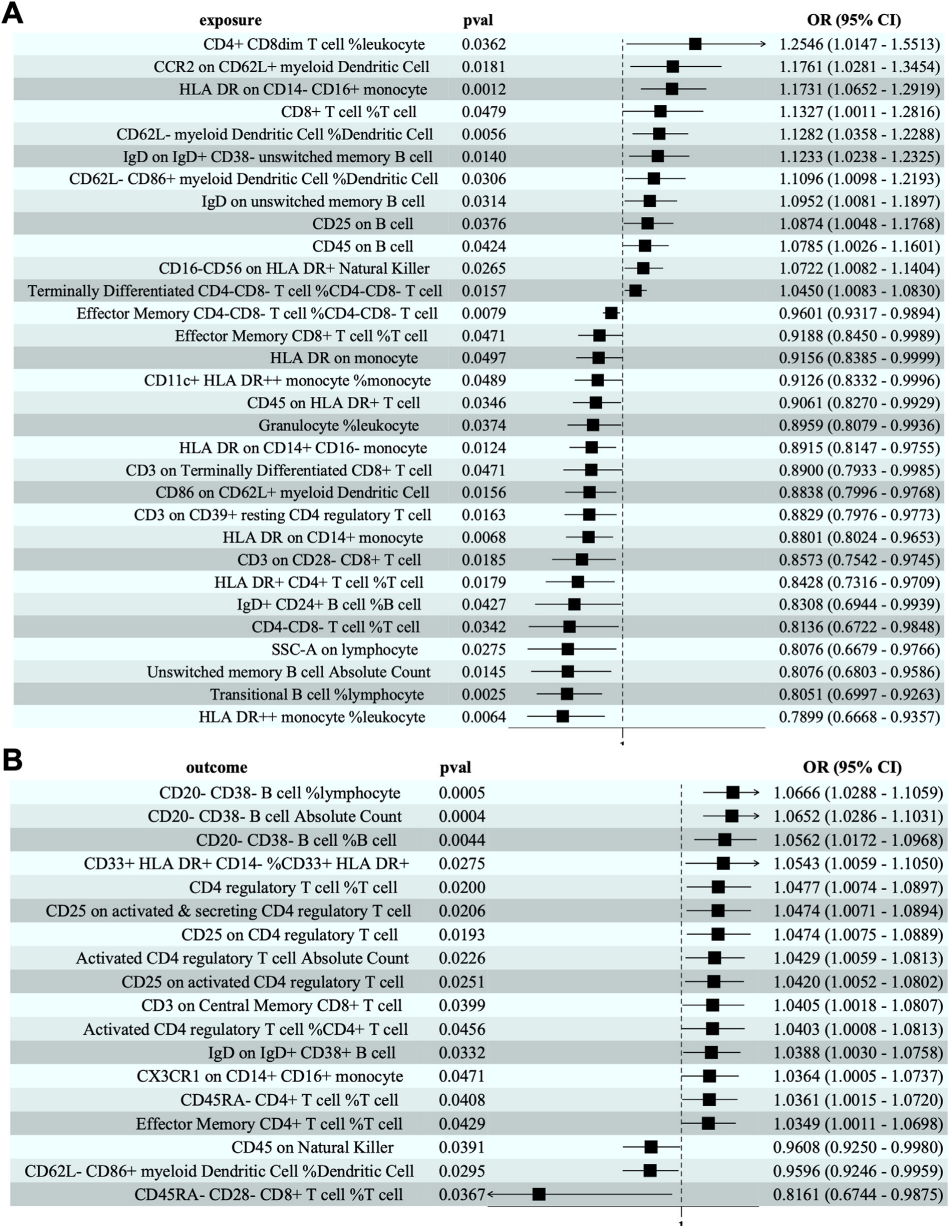
Causal association between immune cells and hypertrophic cardiomyopathy (HCM). **(A)** The forest plot shows the causal association of immune cells with HCM. **(B)** The forest plot displays the causal association of HCM with immune cells. OR, odds ratio; CI, confidence interval.

In examining the reverse causal relationship, HCM was found to significantly impact 18 immune cell types (Fig. 1B). Specifically, HCM served as a protective factor for 15 immune cell types: CD20^−^ CD38^−^ B cell %lymphocyte (OR = 1.0666, *P* = 0.0005), CD20^−^ CD38^−^ B cell absolute count (OR = 1.0652, *P* = 0.0004), CD20^−^ CD38^−^ B cell %B cell (OR = 1.0562, *P* = 0.0044), CD33^+^ HLA DR^+^ CD14^−^ %CD33^+^ HLA DR^+^ (OR = 1.0543, *P* = 0.0275), CD4 regulatory T cell %T cell (OR = 1.0477, *P* = 0.0200), CD25 on activated and secreting CD4 regulatory T cell (OR = 1.0474, *P* = 0.0206), CD25 on CD4 regulatory T cell (OR = 1.0474, *P* = 0.0193), activated CD4 regulatory T cell absolute count (OR = 1.0429, *P* = 0.0226), CD25 on activated CD4 regulatory T cell (OR = 1.0420, *P* = 0.0251), CD3 on central memory CD8^+^ T cell (OR = 1.0405, *P* = 0.0399), activated CD4 regulatory T cell %CD4^+^ T cell (OR = 1.0403, *P* = 0.0456), IgD on IgD^+^ CD38^+^ B cell (OR = 1.0388, *P* = 0.0332), CX3CR1 on CD14^+^ CD16^+^ monocyte (OR = 1.0364, *P* = 0.0471), CD45RA^−^ CD4^+^ T cell %T cell (OR = 1.0361, *P* = 0.0408), and effector memory CD4^+^ T cell %T cell (OR = 1.0349, *P* = 0.0429). Additionally, HCM acted as a risk factor for three immunophenotypes: CD45 on natural killer (OR = 0.9608, *P* = 0.0391), CD62L^−^ CD86^+^ myeloid dendritic cell %dendritic Cell (OR = 0.9596, *P* = 0.0295), and CD45RA^−^ CD28^−^ CD8^+^ T cell %T cell (OR = 0.8161, *P* = 0.0367). Those results indicate a complex interplay between HCM and immune cell dynamics. Moreover, the results from I^2^ and MR-Egger analysis in Table S5 eliminate the possibility of heterogeneity and horizontal pleiotropy. The scatter plots and funnel plots in Figure S5 and Figure S6 demonstrate a broad symmetrical distribution of all the included SNPs, indicating the less likely influence of potential bias on causal associations. The leave-one-out test in Figure S7 suggested that no SNPs could individually affect the causal associations.

This study is the first to confirm bidirectional causal associations between specific immune cell types and HCM through MR analysis. Our findings suggest that alterations in immune cell populations may contribute to the pathophysiology of HCM, while HCM itself can influence immune responses, creating a reciprocal relationship that complicates disease management.

The identification of 31 immune cell types with causal effects on HCM has important clinical implications. These immune cell subtypes may serve as biomarkers for disease diagnosis and progression monitoring, offering potential targets for therapeutic interventions. For instance, the protective role of regulatory T cells suggests that enhancing their levels could mitigate HCM risk,^4^ while strategies aimed at reducing harmful immune populations may also prove beneficial.

Despite the strengths of this MR study, certain limitations warrant consideration. The analysis focused solely on genetic factors, potentially overlooking non-genetic influences such as environmental factors, lifestyle factors, and gene-environment interactions. Additionally, findings derived from European populations may not generalize to other ethnic groups, which could limit the applicability of these results globally, highlighting the need for further studies in diverse cohorts. Lastly, the functional implications of identified immune cell types on HCM have not been validated in animal models.

In conclusion, our bidirectional MR analysis demonstrates significant causal relationships between immune cell types and HCM, underscoring the complexity of immune interactions in the disease's pathophysiology. These insights not only enhance our understanding of HCM but also pave the way for future research aimed at leveraging immune cell profiles for diagnostic and therapeutic purposes.

## Funding

This work was supported by the Program for Youth Innovation in Future Medicine, 10.13039/501100004374Chongqing Medical University, Chongqing, China (No. W0168), the 10.13039/501100005230Natural Science Foundation of Chongqing, China (No. CSTB2022NSCQ-MSX0075), the Joint Project of Chongqing Health Commission and Science and Technology Bureau (China) (No. 2025GDRC006), and Chongqing Education Committee Grant (China) (No. KJQN202300480).

## CRediT authorship contribution statement

**Fang He:** Writing – original draft, Investigation. **Qiangzhong Pi:** Formal analysis. **Jun Yang:** Methodology. **Mengning Wan:** Investigation. **Jiang Yu:** Methodology, Investigation. **Ding Yang:** Investigation. **Yongzheng Guo:** Writing – review & editing, Conceptualization. **Xiaorong Li:** Investigation, Conceptualization.

## Data availability

The datasets presented in this study can be found online. The data supporting the conclusions are included in the article and supplementary materials.

## Conflict of interests

The authors declared no competing interests.
