# Supplementary material for: A causal association between immune cells and hypertrophic cardiomyopathy: A bidirectional Mendelian randomization study
Source: Genes Dis. 2025 Jan 21;12(4):101539. doi: 10.1016/j.gendis.2025.101539 (PMC11994319; doi:10.1016/j.gendis.2025.101539)
Supplement: Multimedia component 1 [file mmc1.docx]

**Supplementary Methods**

**Study design**

This study aimed to determine the causal effects of 731 immune cell types on HCM via a two-sample MR analysis. The IVs were set based on three key assumptions: First, genetic variation is directly associated with exposure. Second, genetic variation is an indecently common confounding factor between exposure and outcomes. Third, genetic variation produces no effects on the outcome via other pathways except for exposure.

**Data sources**

Genome-wide association study (GWAS) summary statistics for immune cells were obtained from the IEU OpenGWAS project (https://gwas.mrcieu.ac.uk/), whereas the GWAS summary statistics for HCM (finngen_R9_I9_HYPERTROCARDMYOP HYPERTROCARDMYOP) were obtained from the Finnish database R9 version (https://storage.googleapis.com/finngen-public-data-r9/). The website was accessed on December 18, 2023. The data included for the analysis in this study were derived from the European population, and the brief information is shown in Table S1. Since informed consent was already obtained for the original study, the ethics committee approval was not required for the present study.

**Instrumental variables (IVs) selection**

To select SNPs from GWAS summary statistics for immune cells, a p-value cutoff of 1×10^−5^ was applied to ensure a direct association between genetic variants and exposure. The bias induced by weak instrumental variables was eliminated by applying a cutoff of F statistics >10. A clumping based on the linkage disequilibrium was performed (R^2^ < 0.001 within a 1,000-kb distance) to ensure that each SNP is independent of each other. The non-0 intercept term of the MR-Egger regression model (P>0.05) was used to remove potential gene pleiotropy. Additionally, SNPs associated with confounders and outcomes were excluded by PhenoScanner (http://www.phenoscanner.medschl.cam.ac.uk/). The relevant SNPs were selected by screening the GWAS summary data of HCM ( R^2^ > 0.8), whereas SNPs that are directly related to HCM were excluded (P<1 ×10^5^). The same steps were applied for reverse causality to screen the associated SNPs.

**Statistical analysis**

An inverse variance weighting (IVW) and MR-Egger were performed to determine the causal association between 731 immune cell types and HCM. This study adopted the IVW analysis as the main method and considered a value of P<0.05 as statistically significant. Cochran’s Q statistic (P<0.05) and I squared (I2>50%) were performed to test the heterogeneity of SNPs. Additionally, the MR-Egger intercept test (P<0.05) was performed to identify potential pleiotropy on causal estimates. Finally, a leave-one-out analysis was performed to observe the impact of each SNP on the bias of causal estimates. All analyses were performed using the Mendelian-randomization package in R 4.1.0 software.

**Supplementary Figures**

Figure S1 Overview of the workflow of MR analysis. 1. Relevance assumption: eligible SNPs for exposure are robustly associated with exposure. 2. Independence assumption: SNPs are not associated with potential confounders. 3. Exclusion restriction: SNPs can only affect Outcomes via Exposure.

Figure S2-1 The scatter plots between different types of immune cells and HCM risk (continue to Figure S2-2).

Figure S2-2 The scatter plots between different types of immune cells and HCM risk (continue to Figure S2-3).

Figure S2-3 The scatter plots between different types of immune cells and HCM risk.

Figure S3-1 The funnel plots between different types of immune cells and HCM risk (continue to Figure S3-2).

Figure S3-2 The funnel plots between different types of immune cells and HCM risk (continue to Figure S3-3).

Figure S3-3 The funnel plots between different types of immune cells and HCM risk.

Figure S4-1 The sensitivity analysis results of causal effects of different types of immune cells on HCM risk (continue to Figure S4-2).

Figure S4-2 The sensitivity analysis results of causal effects of different types of immune cells on HCM risk (continue to Figure S4-3).

Figure S4-3 The sensitivity analysis results of causal effects of different types of immune cells on HCM risk.

Figure S5-1 The scatter plots between different types of immune cells and HCM risk (continue to Figure S5-2).

Figure S5-2 The scatter plots between different types of immune cells and HCM risk.

Figure S6-1 The funnel plots between different types of immune cells and HCM risk (continue to Figure S6-2).

Figure S6-2 The funnel plots between different types of immune cells and HCM risk.

Figure S7-1 The sensitivity analysis results of causal effects of HCM risk on different types of immune cells (continue to Figure S7-2).

Figure S7-2 The sensitivity analysis results of causal effects of HCM risk on different types of immune cells.
