# Supplementary material for: A causal association between immune cells and hypertrophic cardiomyopathy: A bidirectional Mendelian randomization study
Source: Genes Dis. 2025 Jan 21;12(4):101539. doi: 10.1016/j.gendis.2025.101539 (PMC11994319; doi:10.1016/j.gendis.2025.101539)
Supplement: Multimedia component 2 [file mmc2.docx]

**Table S1.** Brief information on the GWAS database

| **Data source** | **Phenotype** | **Sample size** | **Cases** | **Population** | **Adjustment** |
| --- | --- | --- | --- | --- | --- |
| IEU OpenGWAS project | Immune cell | — | — | European | — |
| Finngen Public Data R9(finngen_R9_I9_HYPERTROCARDMYOP) | Hypertrophic cardiomyopathy | — | 1044 | European | — |

Table S2. Some SNPs from the 16,958 selected SNPs for immune cells*.

| **SNP** | **CHR** | **POS** | **EA/OA** | **EAF** | **β*** | **SE** | **P** |
| --- | --- | --- | --- | --- | --- | --- | --- |
| rs114421150 | 4 | 16985307 | A/C | 0.0004 | 2.3728 | 1.2472 | 0.0571 |
| rs114421150 | 4 | 16985307 | A/C | 0.0004 | 2.3728 | 1.2472 | 0.0571 |
| rs10499824 | 7 | 75541772 | T/C | 0.001 | 1.9573 | 0.7717 | 0.0112 |
| rs74827547 | 1 | 54388136 | G/T | 0.0005 | 1.9445 | 0.9904 | 0.0496 |
| rs117690070 | 18 | 11450417 | T/A | 0.001 | 1.7133 | 0.8058 | 0.0335 |
| rs143928375 | 10 | 88482297 | A/G | 0.0005 | 1.7102 | 0.5514 | 0.0019 |
| rs137911637 | 10 | 25016492 | G/A | 0.001 | 1.6467 | 0.7882 | 0.0367 |
| rs139758246 | 16 | 1177020 | C/T | 0.0006 | 1.5962 | 0.9449 | 0.0912 |
| rs138030758 | 20 | 7750823 | T/C | 0.0004 | 1.5663 | 0.6791 | 0.0211 |
| rs146314225 | 5 | 142089851 | C/A | 0.0005 | 1.4894 | 0.9161 | 0.104 |
| ... | ... | ... | ... | ... | ... | ... | ... |
| rs142709521 | 1 | 1042759 | C/G | 0.0007 | -1.1798 | 1.1126 | 0.289 |
| rs191730825 | 1 | 46147567 | G/A | 0.0006 | -1.2023 | 0.9886 | 0.2239 |
| rs191730825 | 1 | 46147567 | G/A | 0.0006 | -1.2023 | 0.9886 | 0.2239 |
| rs191730825 | 1 | 46147567 | G/A | 0.0006 | -1.2023 | 0.9886 | 0.2239 |
| rs138425921 | 1 | 22395458 | T/C | 0.0002 | -1.2472 | 1.7751 | 0.4823 |
| rs73084801 | 12 | 30080934 | T/C | 0.0023 | -1.2769 | 0.7097 | 0.072 |
| rs118006237 | 1 | 100014674 | A/G | 0.0001 | -1.2992 | 2.4541 | 0.5965 |
| rs118006237 | 1 | 100014674 | A/G | 0.0001 | -1.2992 | 2.4541 | 0.5965 |
| rs118006237 | 1 | 100014674 | A/G | 0.0001 | -1.2992 | 2.4541 | 0.5965 |
| rs10107063 | 8 | 122549996 | C/T | 0.004 | -2.4666 | 0.7059 | 0.0005 |

*The detailed data are shown in the attached CSV file named “Table S2 SNPs basic information table”.

Table S3. Some SNPs from the 17831 selected SNPs for HCM*.

| **SNP** | **CHR** | **POS** | **EA/OA** | **EAF** | **β*** | **SE** | **P** |
| --- | --- | --- | --- | --- | --- | --- | --- |
| rs115766721 | 1 | 243548308 | T/G | 0.0006 | 58.48 | 74.33 | 0.4315 |
| rs115766721 | 1 | 243548308 | T/G | 0.0006 | 53.55 | 55.27 | 0.3327 |
| rs187248124 | 15 | 65368655 | C/T | 0.0003 | 38.45 | 76.57 | 0.6155 |
| rs558524282 | 11 | 62609104 | C/T | 0.0076 | 13.88 | 20.54 | 0.4994 |
| rs111857153 | 15 | 63375237 | C/G | 0.0066 | 11.11 | 15.94 | 0.4861 |
| rs30410 | 16 | 79627692 | G/A | 0.9748 | 10.71 | 8.431 | 0.2039 |
| rs147663430 | 15 | 68432551 | C/T | 0.0029 | 9.816 | 22.61 | 0.6642 |
| rs1804972 | 11 | 60658301 | T/C | 0.0339 | 8.229 | 7.332 | 0.2618 |
| rs1804972 | 11 | 60658301 | T/C | 0.0339 | 6.182 | 9.894 | 0.5321 |
| rs11838934 | 13 | 32627358 | G/A | 0.0361 | 5.774 | 9.415 | 0.5397 |
| ... | ... | ... | ... | ... | ... | ... | ... |
| rs111857153 | 15 | 63375237 | C/G | 0.0066 | -11.79 | 21.55 | 0.5845 |
| rs60617331 | 11 | 69715453 | C/T | 0.031 | -12.38 | 10.45 | 0.2359 |
| rs150493778 | 11 | 57967968 | A/G | 0.0003 | -14.38 | 9.244 | 0.1198 |
| rs74566099 | 11 | 45953104 | A/G | 0.0012 | -14.79 | 51.01 | 0.7719 |
| rs144298547 | 15 | 67257392 | G/C | 0.0282 | -18.06 | 8.053 | 0.025 |
| rs145008680 | 15 | 63141760 | C/A | 0.0092 | -18.6 | 18.45 | 0.3136 |
| rs55756397 | 11 | 60285564 | A/G | 0.0063 | -21.21 | 21.94 | 0.3338 |
| rs74566099 | 11 | 45953104 | A/G | 0.0012 | -27.58 | 37.76 | 0.4652 |
| rs150493778 | 11 | 57967968 | A/G | 0.0003 | -49.45 | 69.26 | 0.4752 |
| rs150493778 | 11 | 57967968 | A/G | 0.0003 | -86.2 | 91.11 | 0.3442 |

*The detailed data are shown in the attached CSV file named “Table S3 SNPs basic information table”.

Table S4. Heterogeneity and pleiotropy analysis of the causal effect of immune cells on HCM.

| **Exposure** | **Heterogeneity** | | | **Horizontal pleiotropy** | | |
| --- | --- | --- | --- | --- | --- | --- |
|  | **I²(%)** | **Cochran's Q** | **P-value** | **Egger intercept** | **SE** | **P-value** |
| Effector Memory CD8+ T cell %T cell | 0 | 12.0975 | 0.5197 | 0.0133 | 0.0247 | 0.5988 |
| Unswitched memory B cell Absolute Count | 3 | 17.5399 | 0.4184 | -0.0381 | 0.0367 | 0.3154 |
| IgD+ CD24+ B cell %B cell | 0 | 16.3873 | 0.4966 | -0.0142 | 0.0365 | 0.7015 |
| CD11c+ HLA DR++ monocyte %monocyte | 0 | 11.0546 | 0.7487 | 0.0016 | 0.0222 | 0.9422 |
| CD62L- myeloid Dendritic Cell %Dendritic Cell | 22 | 29.4891 | 0.1646 | -0.0009 | 0.0254 | 0.9729 |
| CD62L- CD86+ myeloid Dendritic Cell %Dendritic Cell | 0 | 13.4829 | 0.7033 | -0.0077 | 0.0230 | 0.7412 |
| HLA DR++ monocyte %leukocyte | 0 | 3.1543 | 0.8704 | -0.0234 | 0.0433 | 0.6091 |
| Effector Memory CD4-CD8- T cell %CD4-CD8- T cell | 0 | 25.1185 | 0.5123 | -0.0039 | 0.0133 | 0.7704 |
| Terminally Differentiated CD4-CD8- T cell %CD4-CD8- T cell | 23 | 31.0356 | 0.1528 | 0.0168 | 0.0163 | 0.3135 |
| Transitional B cell %lymphocyte | 0 | 22.3159 | 0.5604 | 0.0096 | 0.0258 | 0.7133 |
| CD8+ T cell %T cell | 22 | 25.6508 | 0.1777 | 0.0257 | 0.0259 | 0.3331 |
| CD4-CD8- T cell %T cell | 4 | 13.5605 | 0.4055 | 0.0308 | 0.0401 | 0.4565 |
| CD4+ CD8dim T cell %leukocyte | 28 | 16.6481 | 0.1633 | 0.0225 | 0.0458 | 0.6337 |
| HLA DR+ CD4+ T cell %T cell | 0 | 15.4334 | 0.8784 | -0.0247 | 0.0231 | 0.2979 |
| Granulocyte %leukocyte | 1 | 24.2033 | 0.4500 | -0.0042 | 0.0204 | 0.8371 |
| CD25 on B cell | 0 | 8.8061 | 0.9943 | 0.0034 | 0.0209 | 0.8729 |
| IgD on IgD+ CD38- unswitched memory B cell | 0 | 10.8491 | 0.8187 | -0.0218 | 0.0320 | 0.5050 |
| IgD on unswitched memory B cell | 0 | 18.4047 | 0.5608 | -0.0064 | 0.0215 | 0.7680 |
| CD3 on Terminally Differentiated CD8+ T cell | 0 | 17.1565 | 0.6428 | -0.0043 | 0.0340 | 0.8997 |
| CD3 on CD39+ resting CD4 regulatory T cell | 12 | 21.6240 | 0.3034 | 0.0080 | 0.0282 | 0.7791 |
| CD3 on CD28- CD8+ T cell | 0 | 14.0069 | 0.7833 | -0.0184 | 0.0310 | 0.5607 |
| CD16-CD56 on HLA DR+ Natural Killer | 0 | 18.4496 | 0.7327 | -0.0164 | 0.0169 | 0.3441 |
| CD86 on CD62L+ myeloid Dendritic Cell | 0 | 17.3297 | 0.6315 | -0.0086 | 0.0229 | 0.7119 |
| CD45 on B cell | 0 | 9.7791 | 0.7119 | -0.0054 | 0.0204 | 0.7972 |
| CD45 on HLA DR+ T cell | 0 | 13.0185 | 0.7349 | 0.0058 | 0.0276 | 0.8350 |
| HLA DR on CD14- CD16+ monocyte | 0 | 15.4241 | 0.6327 | -0.0185 | 0.0260 | 0.4846 |
| HLA DR on CD14+ CD16- monocyte | 0 | 17.9988 | 0.6491 | -0.0048 | 0.0307 | 0.8769 |
| HLA DR on CD14+ monocyte | 0 | 16.0520 | 0.7134 | -0.0097 | 0.0310 | 0.7590 |
| HLA DR on monocyte | 26 | 21.6348 | 0.1554 | 0.0040 | 0.0361 | 0.9122 |
| CCR2 on CD62L+ myeloid Dendritic Cell | 0 | 6.9701 | 0.9037 | -0.0486 | 0.0368 | 0.2112 |
| SSC-A on lymphocyte | 0 | 13.1928 | 0.6586 | 0.0074 | 0.0436 | 0.8670 |

Table S5. Heterogeneity and pleiotropy analysis for the causal effect of HCM on immune cells.

| **Outcome** | **Heterogeneity** | |  | **Horizontal pleiotropy** | |  |
| --- | --- | --- | --- | --- | --- | --- |
|  | **I²(%)** | **Cochran's Q** | **P-value** | **Egger intercept** | **SE** | **P-value** |
| CD62L- CD86+ myeloid Dendritic Cell %Dendritic Cell | 0 | 18.9574 | 0.7037 | -0.0169 | 0.0149 | 0.2671 |
| CD20- CD38- B cell %B cell | 18 | 29.3425 | 0.2075 | -0.0108 | 0.0149 | 0.4783 |
| CD20- CD38- B cell Absolute Count | 4 | 25.0842 | 0.4012 | -0.0108 | 0.0139 | 0.4438 |
| CD20- CD38- B cell %lymphocyte | 11 | 26.8996 | 0.3092 | -0.0076 | 0.0144 | 0.6040 |
| CD4 regulatory T cell %T cell | 12 | 27.4252 | 0.2850 | 0.0079 | 0.0157 | 0.6174 |
| Activated CD4 regulatory T cell Absolute Count | 0 | 20.3138 | 0.6788 | 0.0081 | 0.0142 | 0.5726 |
| Activated CD4 regulatory T cell %CD4+ T cell | 16 | 28.7290 | 0.2305 | 0.0161 | 0.0152 | 0.2992 |
| CD33+ HLA DR+ CD14- %CD33+ HLA DR+ | 0 | 22.2119 | 0.5075 | 0.0167 | 0.0186 | 0.3782 |
| CD45RA- CD4+ T cell %T cell | 0 | 16.7531 | 0.8590 | 0.0211 | 0.0134 | 0.1275 |
| Effector Memory CD4+ T cell %T cell | 0 | 22.9855 | 0.5207 | 0.0085 | 0.0130 | 0.5208 |
| CD45RA- CD28- CD8+ T cell %T cell | 16 | 28.4256 | 0.2425 | 0.0115 | 0.0764 | 0.8820 |
| IgD on IgD+ CD38+ B cell | 0 | 21.9191 | 0.5841 | -0.0138 | 0.0137 | 0.3267 |
| CD3 on Central Memory CD8+ T cell | 0 | 10.8086 | 0.9903 | 0.0136 | 0.0148 | 0.3667 |
| CD45 on Natural Killer | 0 | 20.7936 | 0.6509 | -0.0017 | 0.0149 | 0.9104 |
| CD25 on CD4 regulatory T cell | 16 | 28.5339 | 0.2382 | 0.0094 | 0.0155 | 0.5497 |
| CD25 on activated CD4 regulatory T cell | 1 | 24.2756 | 0.4459 | -0.0034 | 0.0144 | 0.8176 |
| CD25 on activated & secreting CD4 regulatory T cell | 17 | 29.0346 | 0.2188 | 0.0016 | 0.0157 | 0.9200 |
| CX3CR1 on CD14+ CD16+ monocyte | 0 | 21.0223 | 0.6374 | 0.0153 | 0.0139 | 0.2827 |
